# Miniaturized ultra-wideband filter with independently controlled notch bands for 5.1/6/8 GHz wireless applications

**DOI:** 10.1371/journal.pone.0268886

**Published:** 2022-06-09

**Authors:** Abdul Basit, Muhammad Irfan Khattak, Farid Zubir, Syed Waqar Shah

**Affiliations:** 1 Electrical Department, University of Engineering and Technology, Peshawar, Pakistan; 2 School of Electrical Engineering, Faculty of Engineering, Universiti Teknologi Malaysia, Johor Bahru, Malaysia; UAE University, UNITED ARAB EMIRATES

## Abstract

A novel three-mode step impedance resonator (TSIR) is developed to design a miniaturized multi-pole microstrip planar ultra-wideband (UWB) filter with independently controlled notched bands at different frequencies is presented in this work. The basic UWB filter consists of four L-shaped λ/4 short-circuited stubs operating in the range of i.e. 2.7 to 12.1 GHz with fractional bandwidth (FBW) 127%, and the TSIR is constructed using two T-shaped λ/2 open ended step impedance resonator (SIR) and one λ/4 short-circuited uniform impedance resonator (UIR) is coupled to the basic UWB design in order to achieve the stopbands performance at 5.1 GHz, 6 GHz, and 8 GHz, to supress the unwanted bands of WLAN, Wi-Fi 6E, and X-band satellite communication system, respectively. Commercial full-wave electromagnetic (EM) software HFSS-13 was used to design an ultra-compact structure having size 0.07 λ_g_×0.02 λ_g_ on Rogers RT/Duroid 5880 substrate to justify good agreement between simulated and measured S-parameters.

## 1. Introduction

In today’s world of wireless communication systems, the design of microwave filter has become critical due to increasing demand of advanced communication systems [1, 2]. A review of recent research literature shows that the field of UWB applications have attracted great interest of RF/microwave and academic researchers since the Federal Communications Commission (FCC) licensed frequency band 3.1 GHz to 10.6 GHz for commercial purposes due to its ultra-high-speed data transmission (> 500 Mbit/s), low power dissipation, high selectivity, low insertion loss (IL), and smooth group delay to diminish attenuation in ultra-wideband signal and make it suitable for short distance communication applications [3].

The design of UWB filters with the above specifications is a difficult task for researchers as compared to other types of microwave filters. The problem of larger transceiver size and interference with relatively strong narrow band signals from the WLAN (2.4–2.484, 5.15–5.35, and 5.725–5.825 GHz) or other applications is overcome to introduce the concept of high-attenuation narrow notch bands (NB) capability into the UWB filter [4]. For this several techniques and topologies had been reported in literature in the past few decades for the implementation of UWB filters using different substrate materials. For example, UWB filters were designed by the authors of [5–7] using multilayer liquid crystal polymer technology, multimode resonator (MMR) with shorted step impedance stub, and short-circuited stubs, but the authors did not cover the entire band of UWB (3.1 GHz to 10.6 GHz). Later, different structures of UWB filters were presented in [8–13], using cross shaped resonator, zigzag technique (integrated passive device IPD technology), stepped-impedance open stub resonator, grounded square patch resonator, and composite right-left-handed transmission line (CRLH-TL) resonator. Theses filters exhibit enhanced selectivity and controllable resonant modes/transmission zeros, but the problem of high IL, low FBW, and larger circuit dimensions are associated with the proposed topologies. Recently, UWB filters have been reported with good FBW and IL using MMRs in [14–16], high selectivity using fractal tree stub loaded MMR in [17, 18], respectively. After that several techniques have been reviewed and implemented in the design of UWB filters with multiple stopband characteristics using different resonator topologies i.e., single stopband frequency in [19, 20], dual notch implementation using CPW (coplanar waveguide), step impedance resonator (SIR) technique in [21–23], and triple notch bands implementation with complex structures in [24, 25], respectively.

In this article a compact UWB filter with triple NB’s using TSIR is discussed. The configuration of the proposed resonator has the advantages of the independently controlled stopbands frequencies which make the filter suitable to supress the unwanted signals of the WLAN and X-band satellite communication exists in the UWB range. The first stopband at 5.1 GHz is generated using the upper T-shaped SIR, the second stopband is obtained using the quarter wavelength UIR loaded in the centre of the two T-shaped SIRs, while the third stopband is created using the lower T-shaped SIR couple to the basic UWB filter. Finally, the prototype is fabricated and tested on VNA to validate the theory of proposed BPF. The paper is divided into the following sections, Section II describes the mathematical analysis of the proposed resonator, Section III discusses the cross validation of the stopband frequencies, Section IV describes the filter topology with dimensions, Section V shows the control of stopbands, Section VI explains the experimental and thermotical results, and finally a conclusion is followed.

## 2. Theoretical modelling of the tri-mode resonator and UWB filter

**Fig 1:**
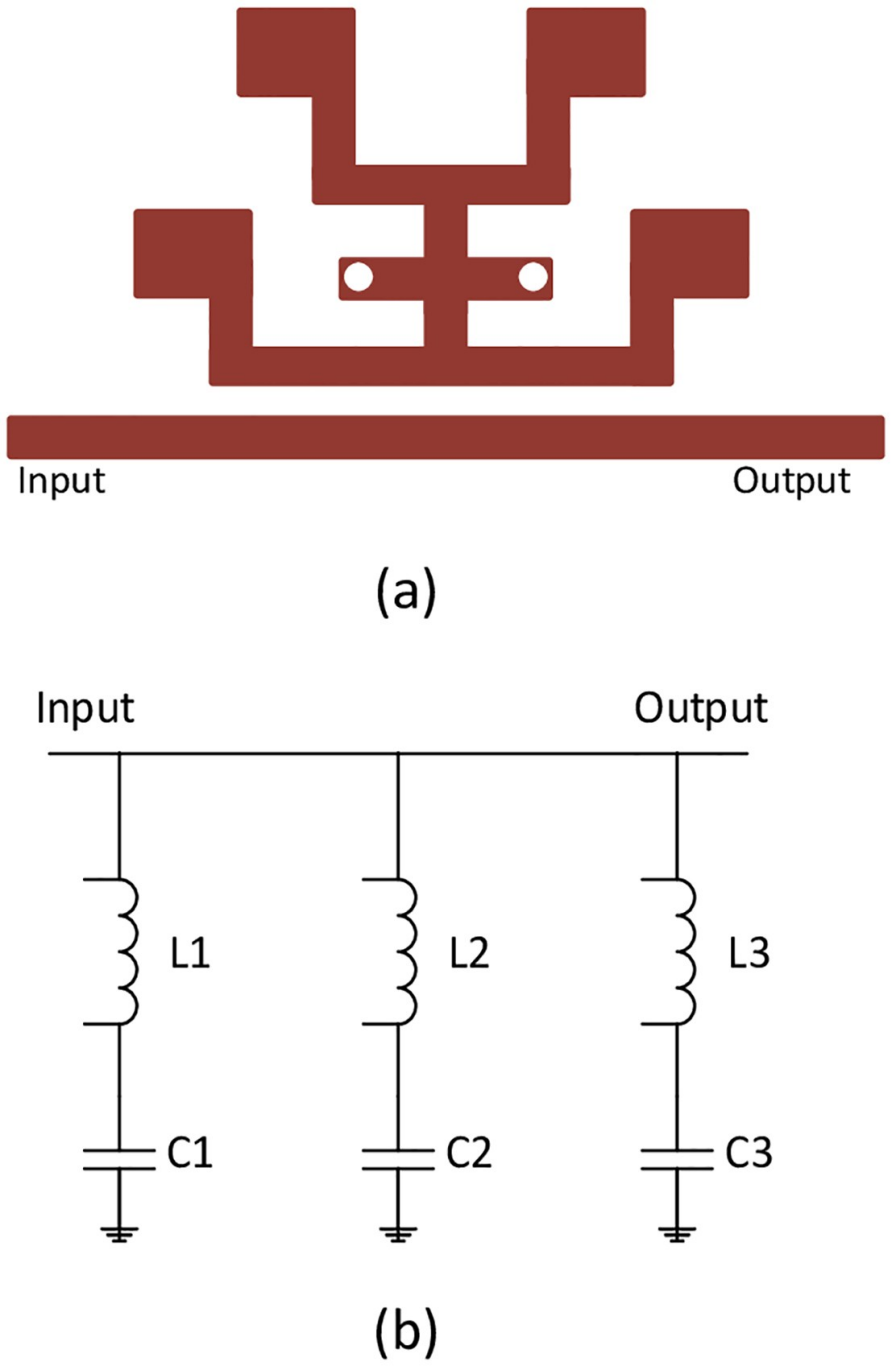
(a) Geometry of the TSIR (b) Equivalent transmission line structure.

The proposed schematic layout of the triple mode resonator along with its equivalent structure is shown in Fig 1. It consists of centrally loaded λ/4 line by tap connecting two T-shaped λ/2 open ended SIR with unequal lengths and widths, θ_i_ (θ_i_ = βL_i_) defines the electrical length of the stubs having the physical length L_i_ and Z_i_ defines the characteristic impedance of the corresponding strips (i = 1, 2, 3, …..). The TSIR is then capacitively coupled to the main transmission line of the basic UWB filter consisting of four λ/4 short-circuited stubs separated by connecting lines with the length of λ/4 and λ/2, respectively, gives three stopbands at different frequencies due to its three modes (two even and one odd mode). All the resonators are folded to reduce the circuit size greatly provided the electrical lengths are fixed.

**Fig 2:**
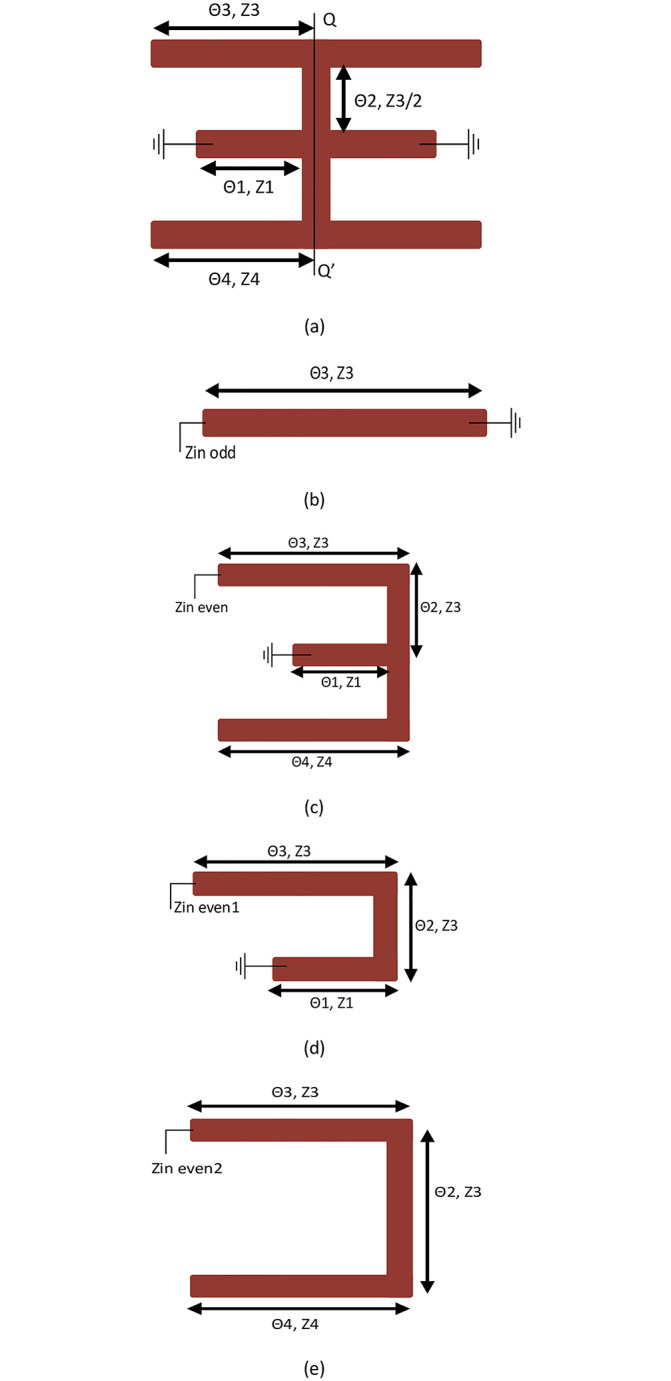
(a) Symmetrical model of the TSIR (b) Odd-mode circuit (c) Even-mode circuit (d) Even mode circuit path I (e) Even mode circuit path II.

The configuration in Fig 2a is symmetrical about ‘QQ’ and could be studied by a famous method called even-odd-mode analysis. Due to its symmetrical nature a total of three modes have been excited, one is odd mode and two are even modes. Fig 2b shows, the equivalent structure of the odd mode analysis consists of one λ/4 resonator with one end shorted while Fig 2c shows, the equivalent structure of the even-mode which is further divided into two resonant circuits, one is λ/4 resonator and the other is λ/2 resonator shown in Fig 2(d) and 2(e), respectively. To simplify the mathematical calculations, assume Z_1_ = Z_3_ = Z_4_, therefore the odd mode input impedance Z_in-odd_ can be deduced as follow [26];

$$Z_{in-}{}_{odd}=jZ_{3}\;\text{tan}\theta_{3}$$ (1)

Put the resonance condition Im(Z_in-odd_) = **∞** in Eq (1), so, the corresponding resonance frequency of the odd mode excitation is obtained as follows;

$$f_{odd}=\frac{(2n+1)c}{4(L_{3})\sqrt{\xi_{eff}}}$$ (2)

Or

$$f_{odd}=\frac{c}{4(L_{2}+L_{b}+L_{4})\sqrt{\xi_{eff}}}$$ (3)

Now the input impedances of the two even modes Z_in-even1_ and Z_in-even-2_ are as follows;

$$Z_{in,even1}=\frac{Z_{1}}{j\text{tan}(\theta_{1}+\theta_{2}+\theta_{3})\sqrt{\xi_{eff}}}$$ (4)

$$Z_{in,even2}=\frac{Z_{1}}{j\text{tan}(\theta_{2}+\theta_{3}+\theta_{4})\sqrt{\xi_{eff}}}$$ (5)

Put the resonance condition Im(Z_in-even_) = **∞** in Eqs (4) and (5), so, the corresponding resonance frequencies of the two even modes excitation are obtained as;

$$f_{even1}=\frac{c}{4(L_{b}+L_{3}{}_{/2}+L_{4}+L_{c}+L_{2/2})\sqrt{\xi_{eff}}}$$ (6)

$$f_{even2}=\frac{c}{2(L_{2}{}_{/2}+L_{3}+L_{b}+L_{1/2}+L_{5}+L_{a}+L_{4})\sqrt{\xi_{eff}}}$$ (7)

From the Eqs 3, 6 and 7, it is verified that the stopband frequencies at 5.1 GHz, 6 GHz, and 8 GHz is totally controlled with the parameters L_1_, L_2_, L_3_, L_4_, L_5_, L_a_, L_b_, and L_c_ of TSIR when placed next to the microstrip transmission line of the basic UWB filter.

## 3. Approach for finding the stopband frequencies

The stopband frequencies for WLAN, Wi-Fi 6E, and X-band satellite communication systems have been adjusted according to the equations derived above from the method of odd-even-mode analysis and the stub lengths chosen. As discussed, the first notch band for WLAN application at 5.1 GHz has been obtained from the odd mode of Fig 2(b) and using Eq (3). By placing the parameter in the denominator of Eq (3), the first notch band at 4.68 GHz is obtained. The second and third stopband for Wi-Fi 6E and XSCS applications at 6 GHz and 8 GHz has been obtained from the even-modes of Fig 2(d) and 2(e) and using Eqs (6) and (7), respectively. So, by putting the stub lengths from Table 2 in the denominator of Eqs (6) and (7), the second and third notch band at 6.33 GHz and 7.62 GHz are obtained. The slight difference in the values of stopband frequencies is due to experimental tolerance of HFSS software. Table 1 shows the theoretical, experimental, and fabricated outcomes of the stopband frequencies of the proposed filter.

**Table 1:** The theoretical, experimental, and fabricated outcomes of the stopband frequencies.

Serial No.	Theoretical result (GHz)	Experimental result (GHz)	Fabricated result (GHz)
1^st^stopband	5.30	5.1	5.16
2^nd^stopband	5.77	6	6.2
3^rd^stopband	7.71	8	8

## 4. UWB filter design with three stopbands

**Fig 3:**
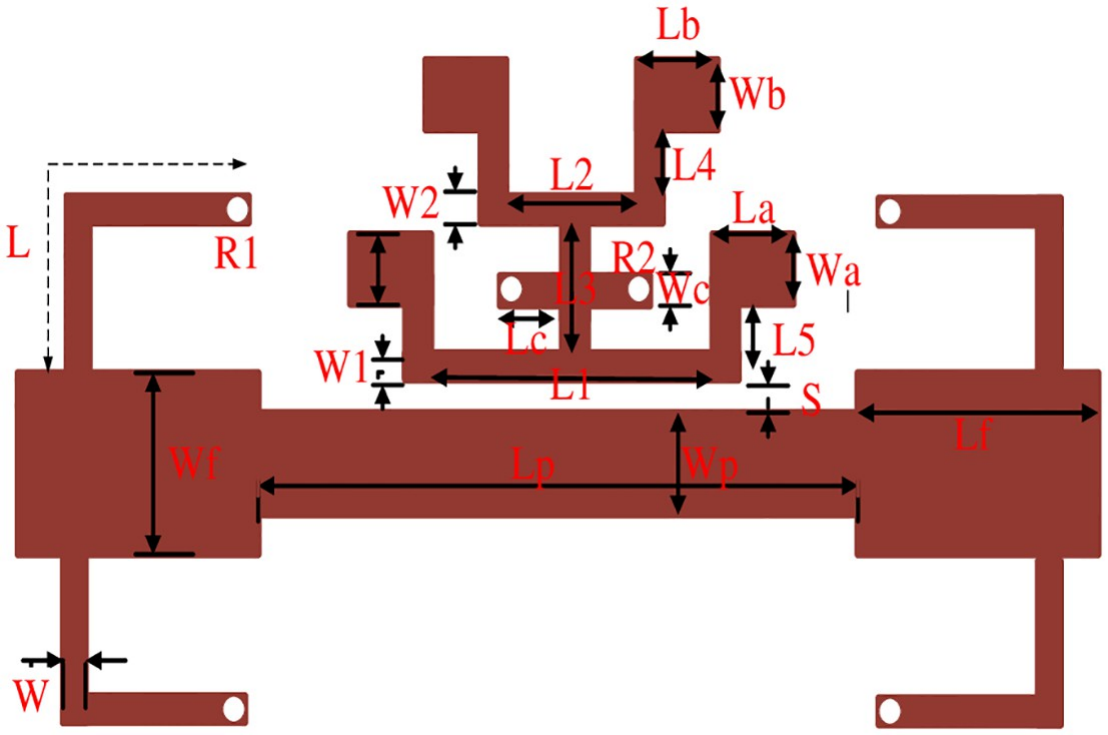
Complete layout of the UWB triple notch filter.

**Fig 4:**
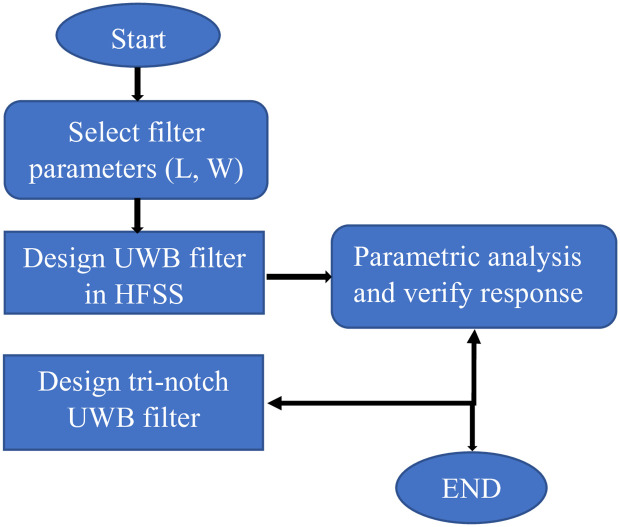
Flow chart for the design of UWB filter with notch bands.

This paper presented a miniaturized UWB filter as shown in Fig 3 with stopband frequencies having overall size 0.07 λ_g_×0.02 λ_g_ (where λ_g_ shows the guided wavelength at first stopband) is fabricated on Rogers 5880 substrate having relative permittivity 2.2 and loss tangent 0.0009, respectively It consists of centrally loaded λ/4 line by tap connecting two T-shaped λ/2 open ended SIR collectively make TSIR which is then coupled to the basic UWB filter consisting of four L-shaped quarter wavelength short-circuited stubs separated by connecting lines with the length of λ/4 and λ/2, respectively. The dimensions in millimetre (mm) of the proposed filter is shown in Table 2 and the design procedure of deriving the UWB filter and the stopband frequencies within it is shown in Fig 4.

**Table 2:** UWB triple notch filter physical parameters in millimetre (mm).

Symbol	value	Symbol	value	Symbol	value
L	9.3	W	0.3	S	0.1
L_1_	5	L_2_	3	L_3_	2
L_4_	1.6	L_f_	9.5	L_a_	1.5
L_b_	1.5	L_c_	1.3	L_p_	15
W_a_	2.4	W_b_	2.4	W_c_	0.3
R_1_	0.2	R_2_	0.1	W_1_	0.2
W_2_	0.2	W_f_	3	W_p_	1.7

## 5. Results and discussion

**Fig 5:**
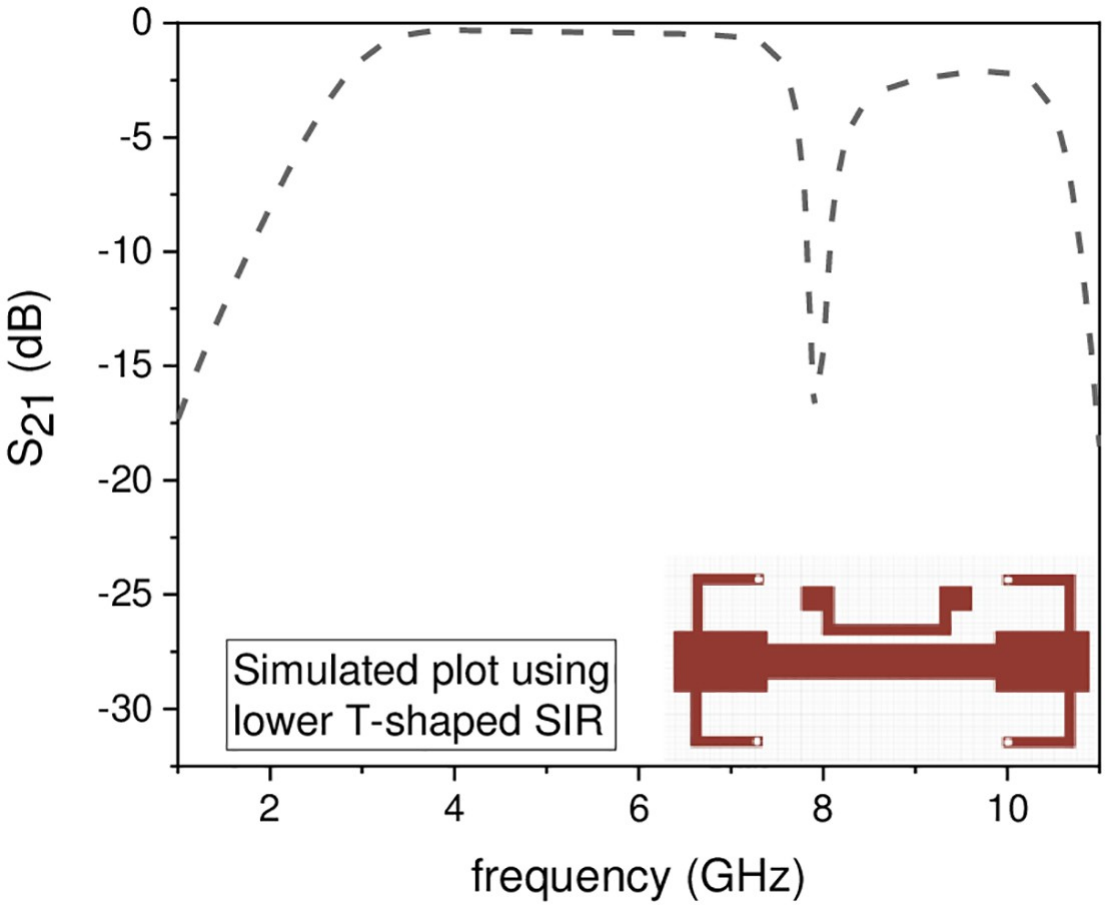
Experimental frequency plot of a single NB using lower T-shaped SIR.

**Fig 6:**
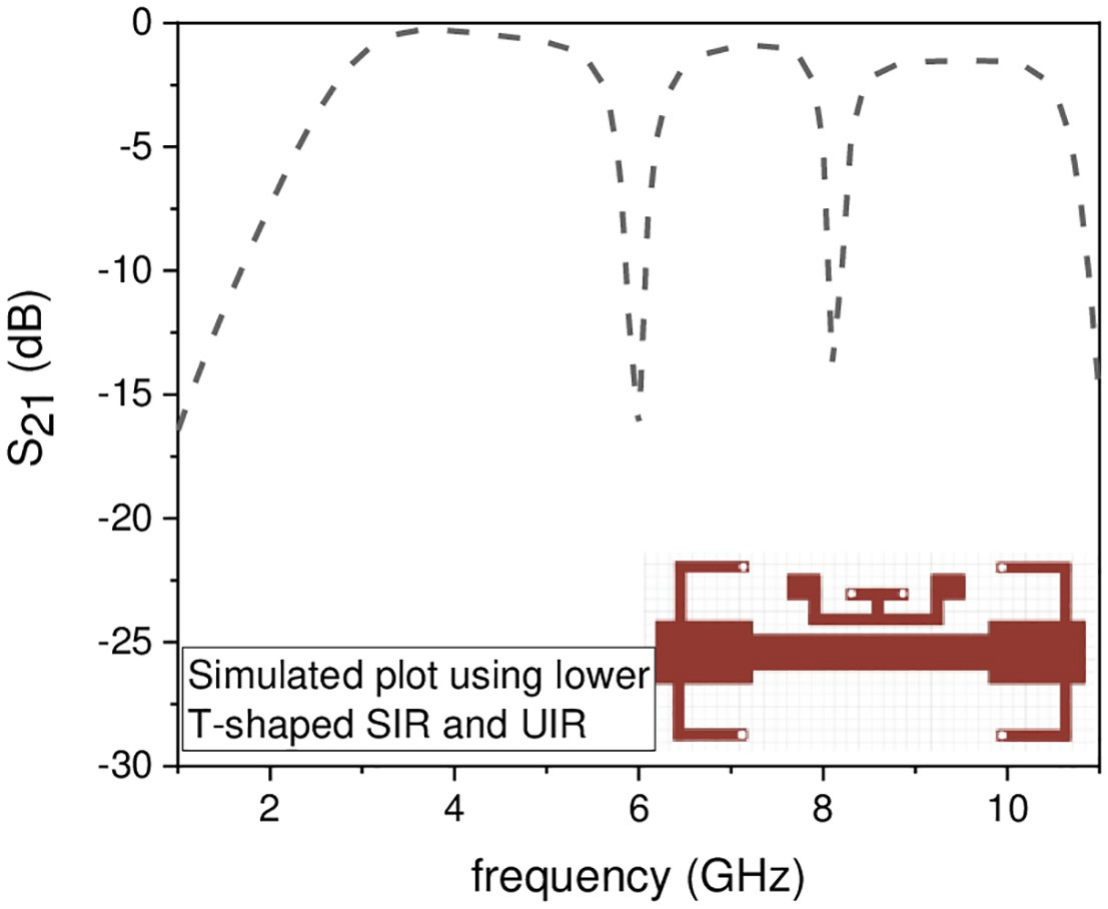
Experimental frequency plot of dual NBs using lower T-shaped SIR and UIR.

**Fig 7:**
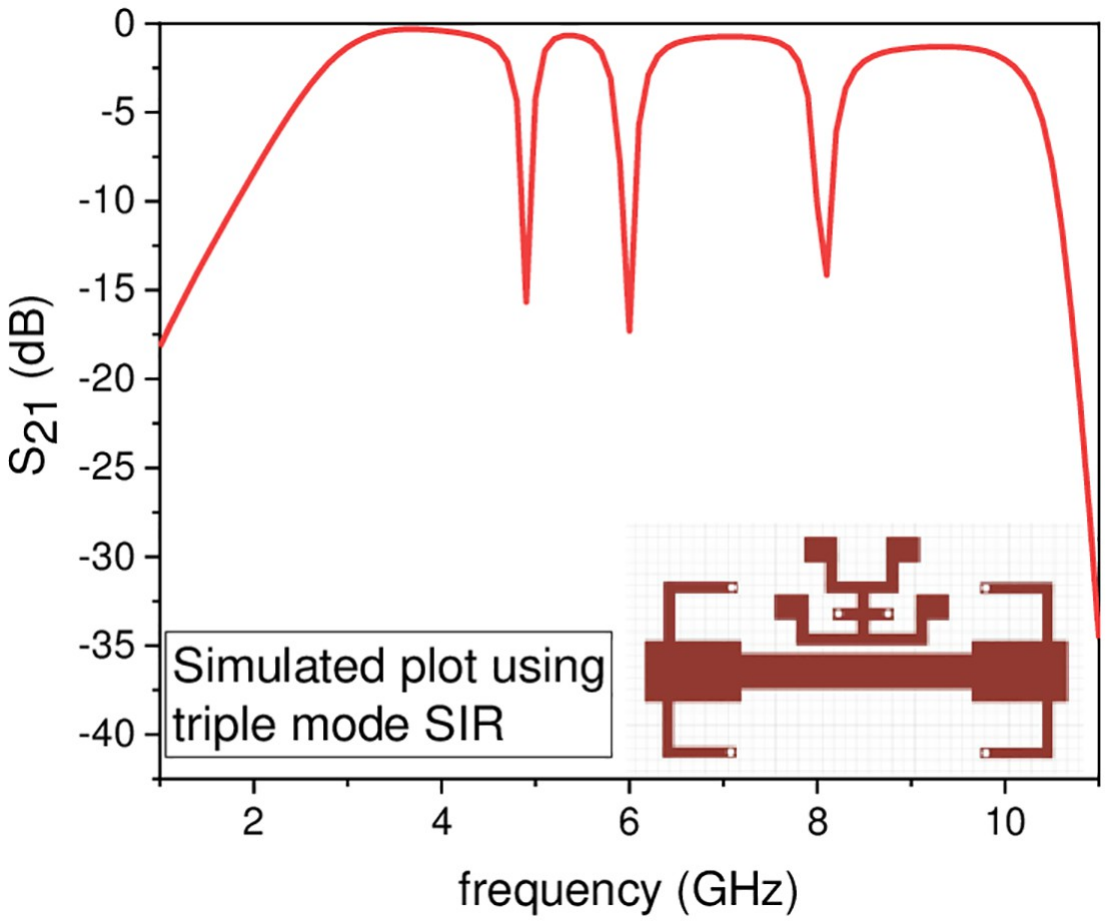
Experimental frequency plot of triple NBs using TSIR configuration.

**Fig 8:**
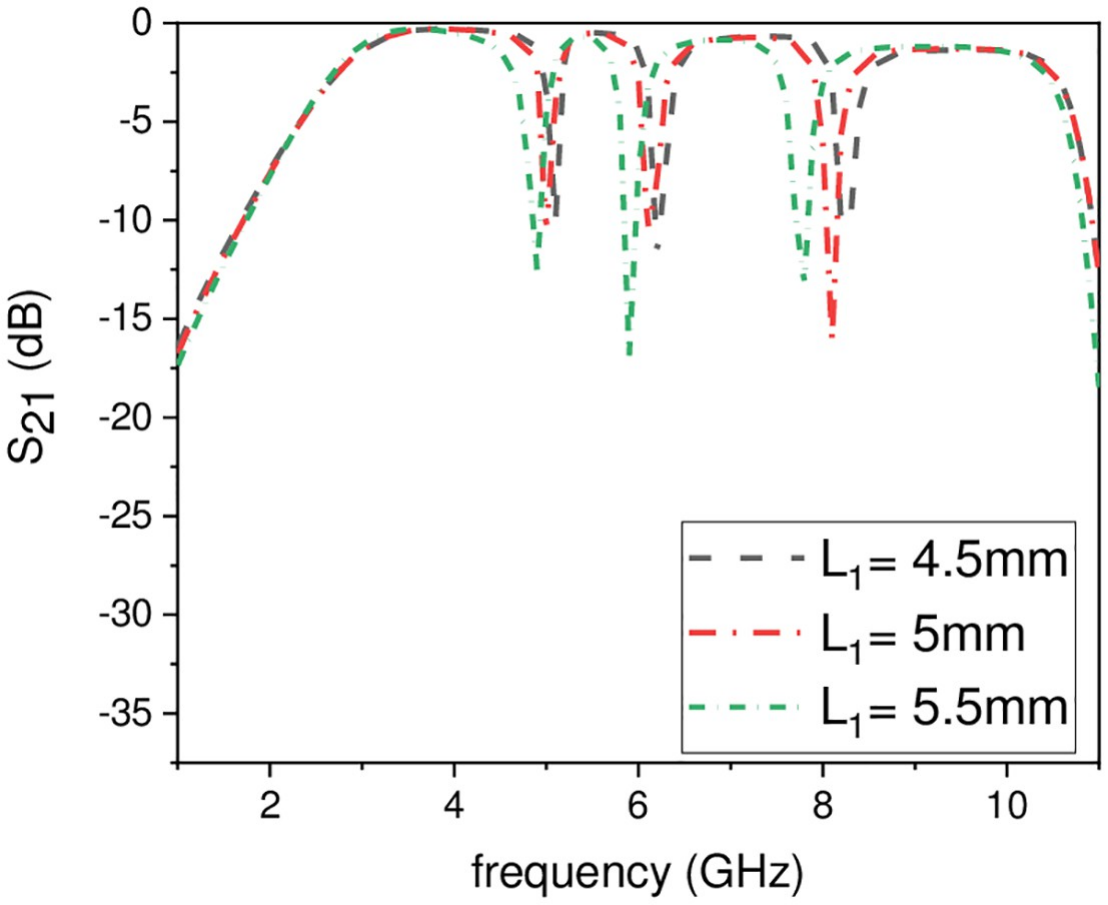
Experimental frequency plot of stopbands by varying L_1_.

**Fig 9:**
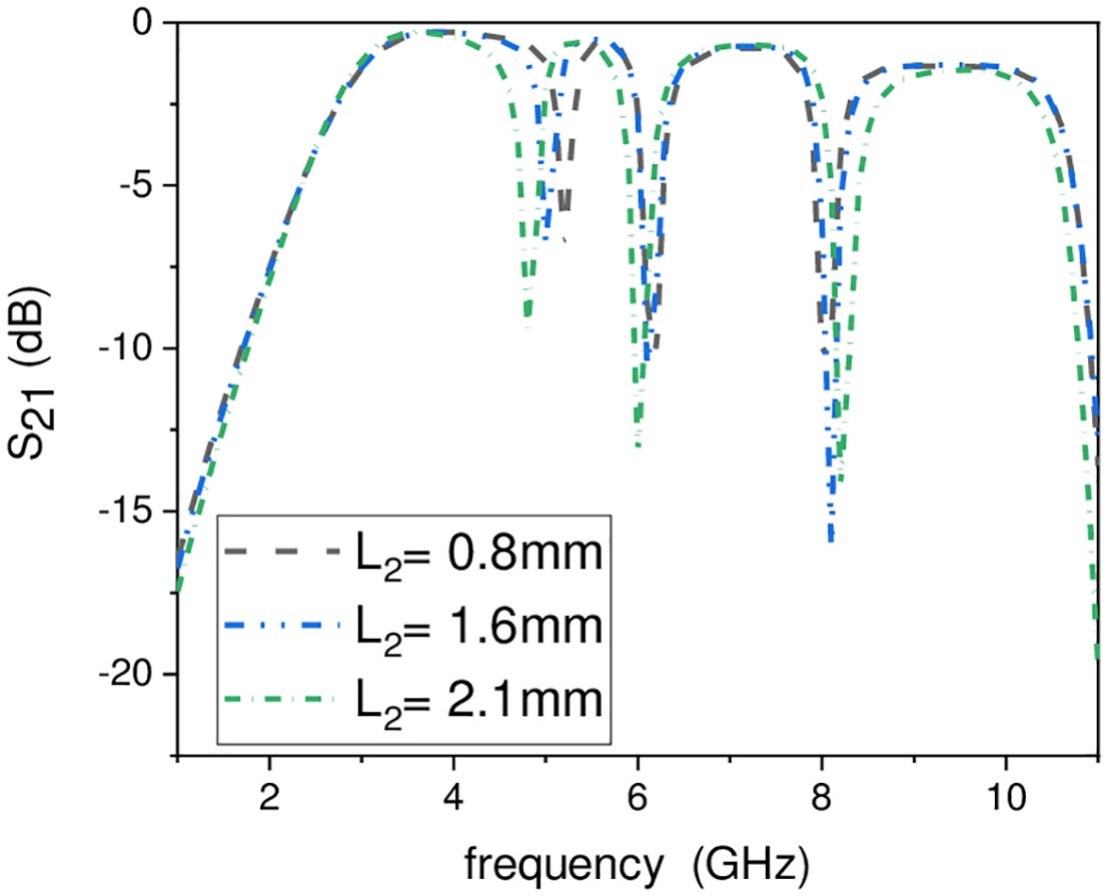
Experimental frequency plot of first NB control by varying L_2_.

**Fig 10:**
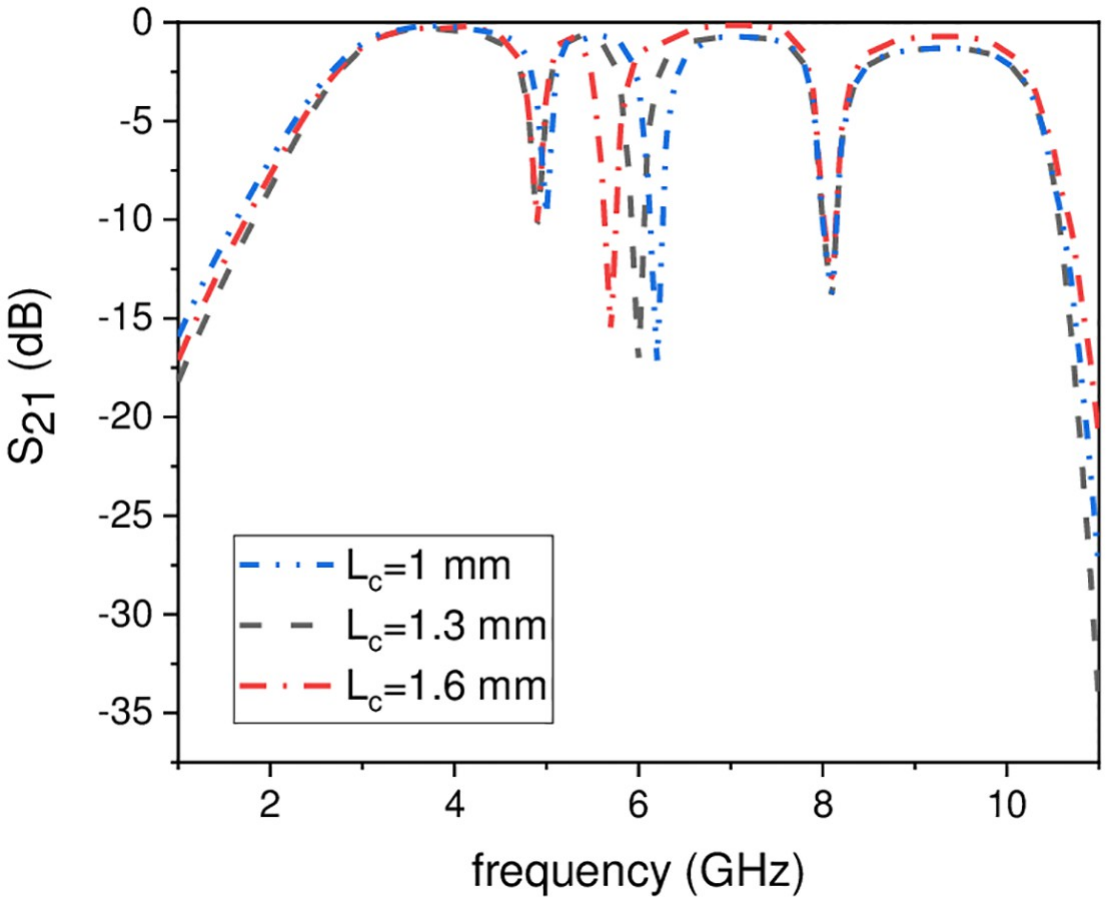
Experimental frequency plot of second NB control by varying Lc.

**Fig 11:**
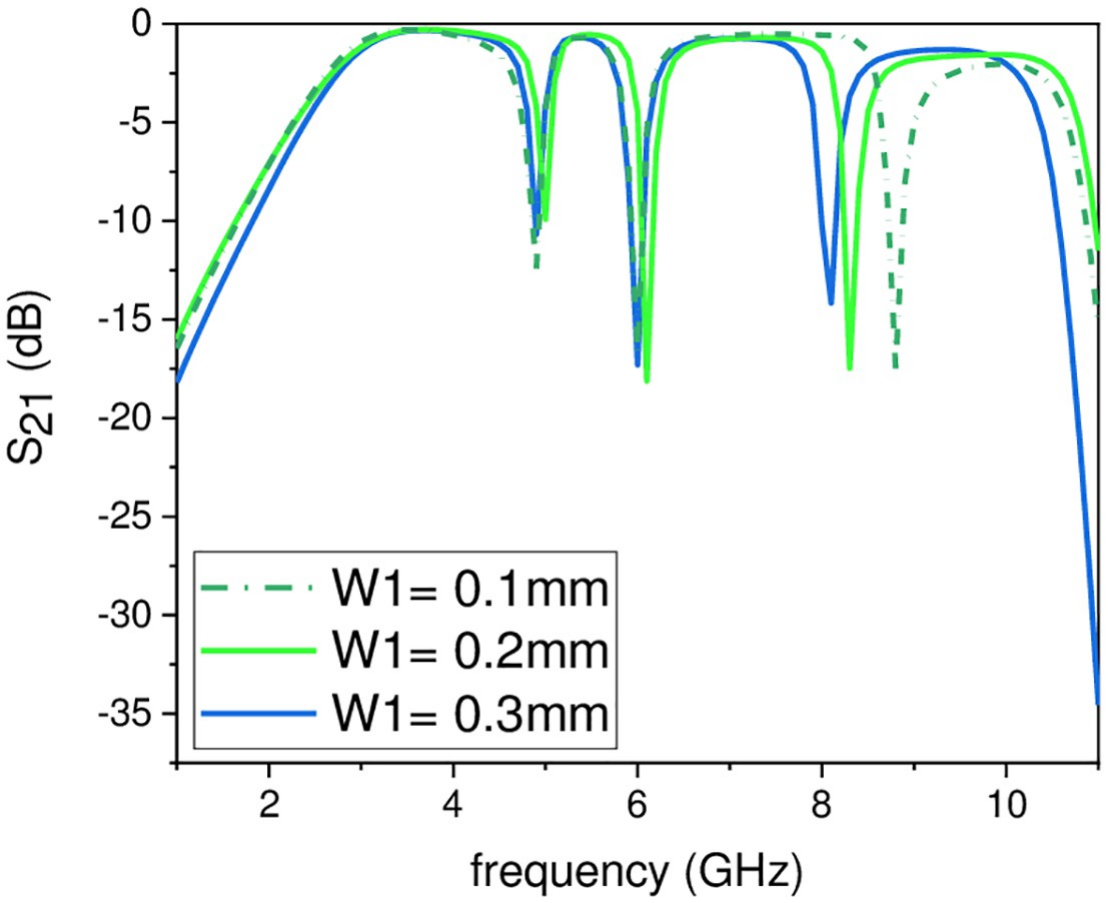
Experimental frequency plot of third NB control by varying W_1_.

In this work, the stopband frequency at 8 GHz is generated using the lower T-shaped SIR as shown in Fig 5, the 6 GHz stopband frequency is obtained using the quarter wavelength UIR loaded in the centre of the two T-shaped SIRs shown in Fig 6, while the stopband at 5.1 GHz is created using the upper T-shaped SIR shown in Fig 7, respectively. The key point of the proposed UWB filter is the super wideband having 3-dB FBW 127% with low IL over the entire UWB range. The other advantage is the control of stopband frequencies independently i.e. by varying the parameter L_1_ from 4.5 mm to 5.5 mm, all the three stopbands are decreases simultaneously as depicted in Fig 8. Fig 9 shows the control of first notch band and by varying the length L_2_, only the first band shifted down from 5 GHz to 4.6 GHz while the other two bands almost constant. Similarly, by changing the stub length L_c_, only the second band will shift down from 6.2 GHz to 5.6 GHz, while the other two bands remain unchanged as shown in Fig 10. When the width W_1_ increases the third notch band decreases from 8.4 GHz to 8 GHz while the remaining two are fixed as depicted in Fig 11. This proves that the proposed filter has the capability to control all the stopbands independently according to the desired frequencies for different wireless applications.

The next important parameter needs to be calculated using Eq (8) is the coupling coefficient (K) which is related to the parameter ‘S’ of Fig 3.

$$k=\frac{f_{2}^{2}-f_{1}^{2}}{f_{2}^{2}+f_{1}^{2}}$$ (8)

**Fig 12:**
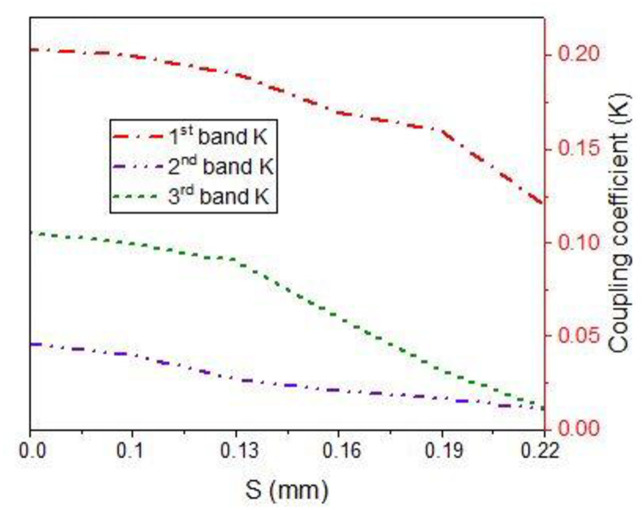
Coupling coefficient “k” against parameter S.

Where f_1_ and f_2_ is the upper and lower stopband frequency. Thus, by increasing the parameter S from 0.1 mm to 0.22 mm, the corresponding coupling coefficient decreases as expected and is plotted in Fig 12, respectively [27].

## 6. Theoretical and experimental results

**Fig 13:**
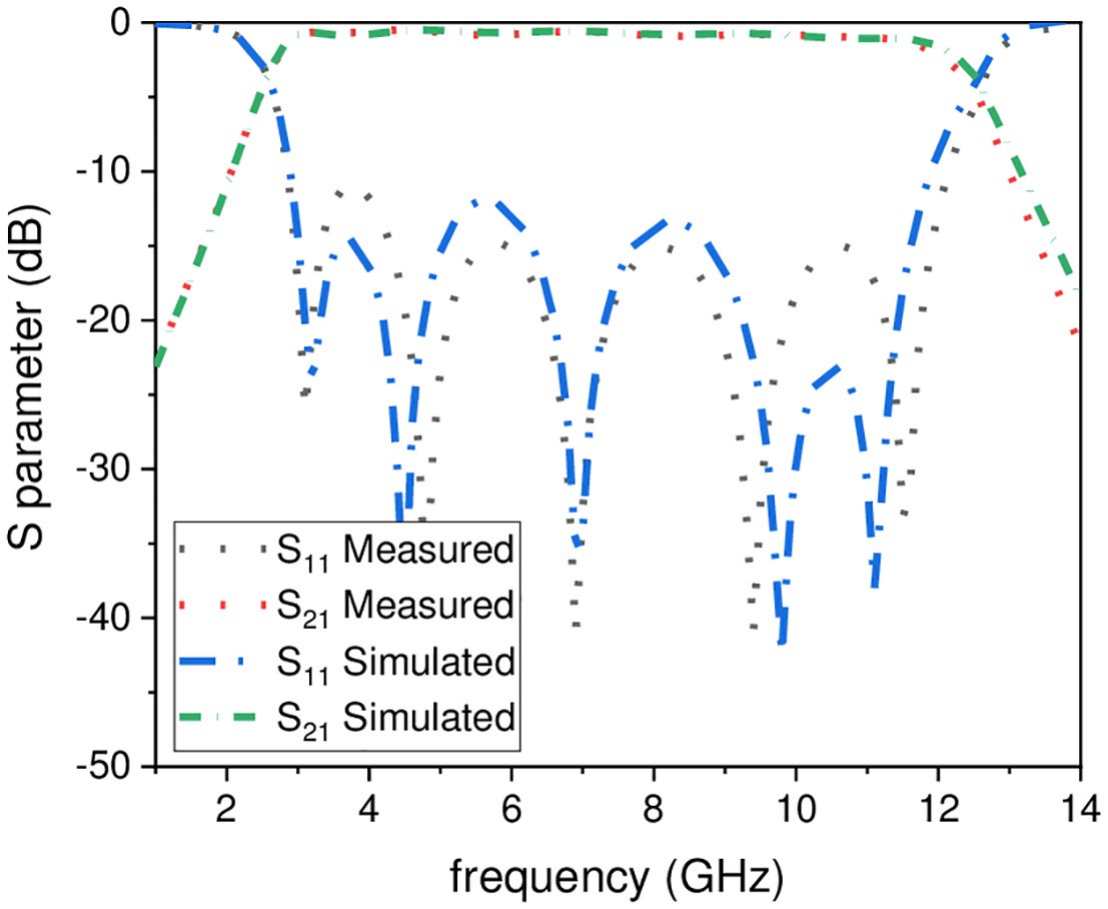
Optimized simulated and measured response of UWB-BPF.

**Fig 14:**
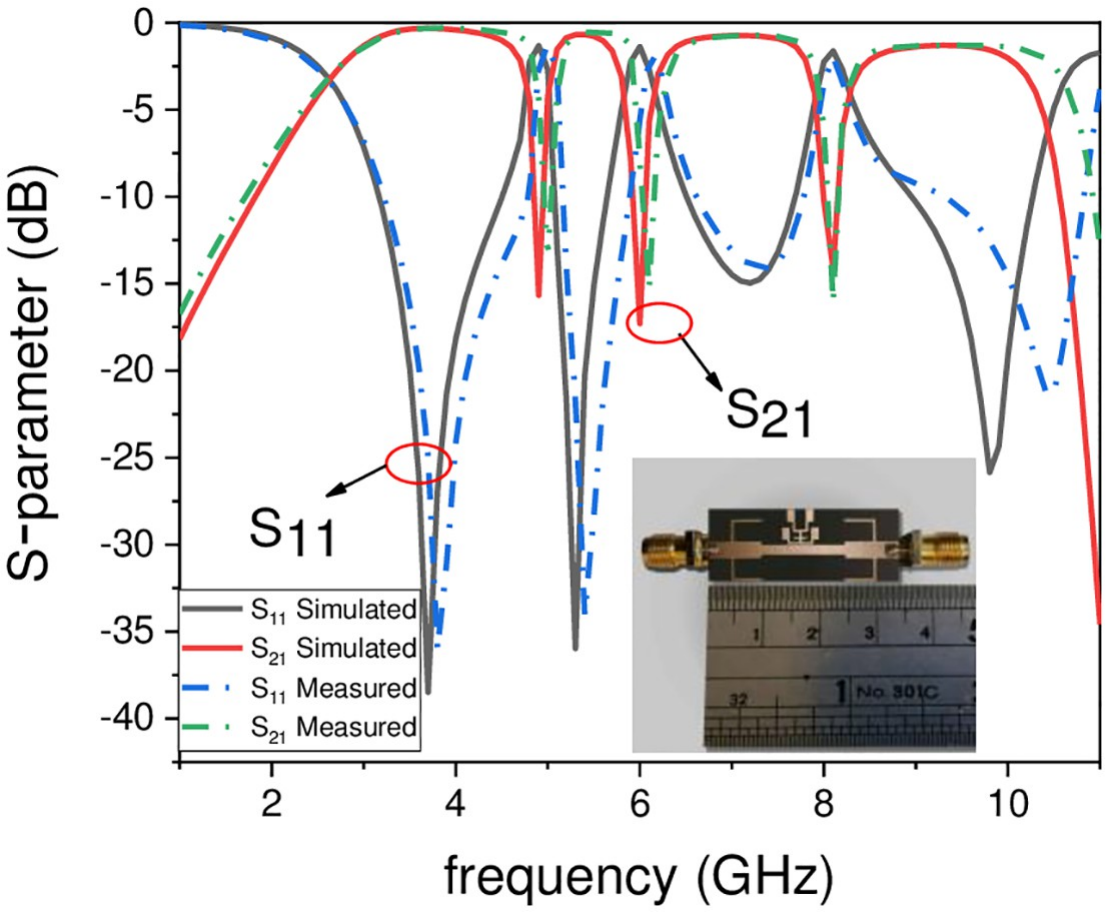
Optimized simulated and measured outcomes of the proposed filter.

A compact UWB filter with independently controlled stopbands using a novel TSIR is discussed in this paper. The proposed UWB filter has a super wideband starting from 2.7 GHz to 12.1 GHz with the 3-dB FBW 127%, central frequency (CF) 7.4 GHz, and 0.1 dB IL over the entire band as depicted in Fig 13. This bandwidth covers the basic requirements of the UWB range authorized by FCC in 2002 having fractional bandwidth not less than 109% [1]. To supress the unwanted signal in the UWB range i.e., WLAN, Wi-Fi 6E, and X-band satellite communication system, a triple mode SIR is embedded in the UWB filter to introduce the stopbands at 5.1 GHz, 6 GHz, and 8 GHz, with rejection level greater than -15 dB and insertion loss lower than -1.3 dB for all the three stopbands and is tabulated in Table 3. Fig 14 shows the simulated and measured frequency plots of the UWB filter with notch band frequencies which clearly reveals that the proposed filter is a suitable candidate for many broadband wireless communication systems. The slight deviations in simulated and measured results are due to the connector losses and the finite substrate. To verify the compactness and super wideband, the proposed filter is compared with the state-of-the-art designs published in reputed journals/conferences as shown in Table 4, respectively.

**Table 3:** Experimental outcomes of the proposed tri notch filter.

NB (GHz)	Absolute BW (MHz)	3 dB FBW (%)	Rejection level
5.1	300	6	16
6	550	6.5	18
8	500	6.13	15

**Table 4:** Comparison of this work with other reported literature.

Ref. No	Passband (GHz)	FBW %	IL dB	NB (GHz) / Attenuation (dB)	Size (*λ*_g_× *λ*_g_)
[6]	3.4–10.7	106	0.7	Not available	0.48×0.12
[7]	3–10	107.7	0.6		0.27 × 0.2
[11]	3.1–10.6	115	NA		0.38×0.30
[13]	2.85–10.67	105	<3		0.63×0.53
[16]	3.1–10.6	110.2	0.35		0.39×0.11
[15]	3.1–10.6	109	<0.5		0.74×0.67
[18]	3.6–10.4	103.9	> -1.5		0.52×0.43
[17]	3.7–9.6	106.2	<1		0.48×0.40
[14]	2.94–10.39	111.6	0.5		0.76×0.47
**UWB filter with notch bands implementation**					
[8]	3–10.5	111	1.2	4.3, 9.1/ 28, 19	0.57×0.54
[9]	3.0–10.3	92	2	5.8, 8/ >15	0.89 × 0.36
[20]	3.1–11	112	0.66	6/ 35	0.41 × 0.26
[21]	3–10.9	110	NA	5.96, 8.15 />15	0.77 × 0.38
[23]	3.58–10.07	95.1	<1.2	5.53, 8.1/ >15	0.24 × 0.61
[24]	3.25–10.73	106.6	0.52	5.6, 6.4, 8.03/ >19	1.04×0.66
[25]	3.09–10.61	109	<1.25	3.6, 5.3, 8.4/ >16	0.74×0.61
**This work**	**2.7–12.1**	**127**	**<1**	**5.1, 6, 8/ >15**	**0.07 × 0.02**

## 7. Conclusions

In this letter, a simple design approach has been presented to design an ultra-compact UWB filter with notch bands features having size 0.07 λ_g_ × 0.02 λ_g_ using T-shaped tri mode SIR resonator and L-shaped quarter wavelength resonator. Compared with the recent published filters the resulted UWB filter significantly improves the 3 dB fractional bandwidth of 127% with low IL less than 0.85 dB. It has been proven that the three notch bands can be introduced into the passband of the filter allowing the high rejection of spurious signal for WLAN, Wi-Fi 6E, and X-band satellite communication systems at 5.1 GHz, 6 GHz, and 8 GHz, with sharply rejection level greater than -15 dB and good overall out of band performance. These characteristics make the proposed structure a suitable commercial product for many broadband wireless communications systems.

## Supporting information

S1 Data (XLSX)
